# Enhancing collaboration between academia and industry in kidney disease research

**DOI:** 10.1093/ndt/gfae272

**Published:** 2024-11-28

**Authors:** Robert J Unwin, Benjamin Challis, Iain MacPhee, Pernille B L Hansen, Rachel Jones, Alan Salama, Jonathan Barratt

**Affiliations:** AstraZeneca Biopharmaceuticals R&D, Early Cardiovascular, Renal and Metabolism (CVRM), Cambridge, UK and Gothenburg, Sweden; UCL Centre for Kidney and Bladder Health, University College London, London, UK; AstraZeneca Biopharmaceuticals R&D, Early Cardiovascular, Renal and Metabolism (CVRM), Cambridge, UK and Gothenburg, Sweden; AstraZeneca Biopharmaceuticals R&D, Early Cardiovascular, Renal and Metabolism (CVRM), Cambridge, UK and Gothenburg, Sweden; AstraZeneca Biopharmaceuticals R&D, Early Cardiovascular, Renal and Metabolism (CVRM), Cambridge, UK and Gothenburg, Sweden; Department of Medicine, University of Cambridge, Cambridge, UK; UCL Centre for Kidney and Bladder Health, University College London, London, UK; John Walls Renal Unit, University of Leicester, Leicester, UK

In recent years, medical research and drug development have seen remarkable progress in various fields, but nephrology continues to lag behind. Kidney disease research faces numerous challenges, with drug approvals for nephrology averaging less than 1% of all new approvals in the past 5 years [1]. Addressing these gaps and improving outcomes for kidney disease patients require more effective collaboration between academia and the pharmaceutical industry, whose resources and expertise are essential to overcome the barriers slowing nephrology's advancement.

Kidney diseases are uniquely complex, marked by high heterogeneity across patient populations. Clinical manifestations, disease progression and responses to treatment vary widely, complicating the identification of effective therapeutic targets. In addition, the limited translatability of animal models, the slow progression of kidney diseases and the lack of reliable biomarkers compound the challenge, making it difficult to design and complete clinical trials within reasonable timelines [2]. Unlike fields such as oncology, where biomarkers have revolutionized clinical trials, nephrology still relies heavily on markers like albuminuria and proteinuria, which lack precision in predicting treatment outcomes and disease progression. The absence of more sophisticated biomarkers makes it harder to identify patients likely to benefit from new treatments, ultimately slowing trial efficiency and drug development.

To accelerate nephrology research and drug development, a well-defined framework is essential to facilitate collaboration between academia and industry. Such a framework must address significant barriers, including intellectual property (IP) rights, differences in institutional priorities, cultural discrepancies and the crucial role of patient advocacy groups. The financial risks associated with drug development in nephrology are considerable (see Table 1) due to the high failure rates and prolonged timelines. Therefore, industry often takes a cautious approach, investing selectively. Collaboration models that share financial burdens, streamline treatment target identification and testing, and accelerate timelines are needed to make nephrology research viable and effective.

**Table 1:** These estimates illustrate the substantial financial investment required at each stage of drug development for a single drug; the costs are particularly significant in nephrology, where the complexity of kidney diseases necessitates extensive and rigorous testing to ensure that new treatments are both safe and effective.

Phase of drug development	Estimated cost	Key activities
Discovery and preclinical	$100–$500 million	Target identification, lead optimization, animal testing
Phase 1 (safety trials)	$100–$200 million	Initial testing in humans to assess safety and dosage
Phase 2 (efficacy trials)	$300–$500 million	Testing in a larger group to evaluate efficacy and side effects
Phase 3 (large-scale trials)	$500 million–$1 billion	Large-scale testing for efficacy and monitoring of adverse reactions
Regulatory review and approval	$50–$100 million	Submission of data to regulatory bodies, responding to inquiries
Post-marketing surveillance	$50–$200 million	Long-term monitoring of a drug's effect in the general population

Innovation in drug development often begins with foundational academic research, where breakthroughs sometimes arise serendipitously, as in the case of sodium-glucose cotransporter 2 inhibition and glucagon-like peptide-1 receptor agonism for chronic kidney disease (CKD) [3, 4]. Academic institutions are key to identifying novel targets and understanding disease mechanisms, but translating these discoveries into treatments requires the resources and expertise of the pharmaceutical industry. Successful partnerships hinge on clear frameworks that align shared goals, address potential conflicts of interest, and balance both public health and commercial priorities.

To achieve this, several practicable strategies can help to foster collaboration:

- Establish collaborative research committees: these committees, comprising representatives from academia, industry and patient advocacy groups, can provide oversight and guide projects from inception to completion. Their diverse perspectives on trial design, research objectives and patient-centred outcomes can improve clinical trial efficiency by identifying relevant biomarkers early, enabling patient stratification and addressing quality-of-life outcomes as potential trial endpoints. Involving patient advocacy groups ensures studies remain relevant to broader public health needs.
- Address IP rights: IP disputes frequently hinder collaborative research, as differing priorities can create conflicts over ownership. Pre-negotiated IP-sharing agreements provide incentives for both parties without compromising academic freedom or commercial interests. Models that allow shared IP ownership and flexible licensing terms could permit academia to retain access for ongoing research, while industry gains exclusive commercialization rights. AstraZeneca's Open Innovation platform, which allows access to its compound library, and its collaboration with the UK Medical Research Council (MRC), exemplify early steps toward open, mutually beneficial IP practices [5].
- Ensure long-term funding and sustainability: nephrology research requires dedicated, sustained funding. While industry–academic collaborations have increased, many focus on short-term projects. A survey by the Association of the British Pharmaceutical Industry indicates a notable rise in industry investment in academia, including collaborative training opportunities within the Horizon Europe framework [6]. Extending these partnerships through public–private initiatives, similar to the Innovative Medicines Initiative in Europe, could help academia and industry share financial and operational responsibilities, moving beyond project-specific investments. Such partnerships should also aim to develop more diverse clinical endpoints that capture real-world patient experiences, ensuring that research outcomes reflect the complexities of kidney disease across diverse demographics. The NURTuRE (National Unified Renal Translational Research Enterprise) adult CKD cohort in the UK offers a valuable model by integrating patient involvement in study design, providing clinical and biomarker data, and creating a ready pool of patients and investigators for clinical trials [7].
- Create dedicated biomarker development centres: a persistent lack of specific biomarkers impedes progress in nephrology. Dedicated centres, co-funded by academia and industry, could prioritize discovering and validating biomarkers needed for interim analyses and adaptive trial designs, allowing clinical trials to be adjusted based on ongoing results [8]. By improving patient selection and facilitating adaptive trial designs, these centres can help reduce study costs and timelines. Such centres could also address nephrology's limitations in traditional histological analysis with the application of machine learning [9] and combined with a multi-omics approach [10]. For example, while immunoglobulin A (IgA) nephropathy and IgA vasculitis may appear similar histologically, they differ in disease progression and treatment response, underscoring the need for biomarkers that capture disease nuances and predict therapeutic efficacy more reliably [11].

However, despite such initiatives, significant barriers remain that must be addressed to make collaborations effective:

- IP and ownership disputes: academic and commercial interests often clash when research priorities and revenue potential diverge. Transparent, fair IP agreements are essential to promote a collaborative environment. Revenue-sharing models can align incentives, granting industry necessary commercialization rights while allowing academia to continue valuable research.
- Divergent goals and institutional priorities: academic institutions prioritize scientific exploration and publication, while industry focuses on product development and profitability. Structured agreements that explicitly define contributions, timelines and outcomes can help align priorities, reduce conflicts and ensure mutually beneficial outcomes.
- Contrasting cultural approaches: academia typically emphasizes methodical investigation, while industry values expediency and results. Joint training programmes and internships could bridge this gap by exposing researchers to both environments. The MRC and AstraZeneca fellowships, which blend academic and industry training, exemplify schemes that foster interdisciplinary expertise, equipping researchers to contribute effectively in either setting.
- Managing industry influence in research: industry's focus on profitable ventures poses risks, as it can skew research agendas toward high-return treatments rather than addressing broader public health needs. Transparent research goals, public oversight and patient advocacy can help ensure that collaborations serve public health priorities. Patient advocates provide valuable insights on quality of life, treatment accessibility and symptom management, often overlooked in traditional research. Involving patient advocates in trial design and biomarker selection not only enhances the relevance of studies but also builds public trust.

Creating a sustainable research model also requires developing a workforce proficient in both academic and commercial research environments. Programmes offering dual roles or fellowships, like the MRC and AstraZeneca initiative, allow young researchers to gain hands-on experience across sectors, equipping them to advance drug development in complex fields like nephrology. Established clinical scientists could also benefit from industry secondments, exchanging knowledge and experience that ultimately benefit both academia and industry. However, making it possible to balance clinical duties with industry experience will be crucial in cultivating the next generation of clinician-scientists, whose cross-disciplinary expertise will be essential for tackling the unique challenges in kidney disease research.

Balancing collaboration between academia and the pharmaceutical industry is critical to advancing drug development in nephrology. Pharmaceutical companies face challenges beyond scientific discovery, such as technical, operational and financial hurdles. Long-term partnerships between academia and industry can yield substantial, enduring benefits. By committing to sustained collaboration and addressing the barriers discussed, academia and industry can ensure resources are used efficiently, advancing research aligned with both scientific and patient needs. This approach is vital in nephrology, where disease complexities demand a concerted, well-coordinated effort focused on patient benefit.

In ‘grasping the nettle’ and forging these essential partnerships, the future of kidney disease research holds great promise. As researchers harness the power of multi-omics and artificial intelligence applications, the classification of renal diseases may evolve, encouraging a shift away from what might be described as CKD treatments toward disease-specific drug targets. By identifying precise molecular and genetic markers, this approach could lead to targeted treatments that can address the subtleties of individual kidney diseases. Such advancement would be a major step forward in kidney research, aligning new drug development more closely with the diverse and complex realities of kidney disease.

With long-term, transparent collaborations, academia and industry can accelerate innovation, overcome current obstacles and ultimately transform kidney disease treatment—ensuring patients benefit from every advance made in nephrology research.
